# T cell mediated cerebral hemorrhages and microhemorrhages during passive Aβ immunization in APPPS1 transgenic mice

**DOI:** 10.1186/1750-1326-6-22

**Published:** 2011-03-09

**Authors:** Melanie Meyer-Luehmann, J Rodrigo Mora, Matthew Mielke, Tara L Spires-Jones, Alix de Calignon, Ulrich H von Andrian, Bradley T Hyman

**Affiliations:** 1 MassGeneral Institute for Neurodegenerative Disease, Massachusetts General Hospital, Harvard Medical School, Department of Neurology, Alzheimer's Disease Research Laboratory, 02129 Charlestown, MA USA; 2 German Center for Neurodegenerative Diseases (DZNE), 80336 Munich, Germany; 3 Adolf-Butenandt-Institute, Biochemistry, Ludwig Maximilians-University, 80336 Munich, Germany; 4 Immune Disease Institute & Department of Pathology, Harvard Medical School, Boston, MA 02115, USA; 5 Gastrointestinal Unit, Massachusetts General Hospital, Harvard Medical School, Boston, MA 02114, USA

## Abstract

**Background:**

Immunization against amyloid-β (Aβ), the peptide that accumulates in the form of senile plaques and in the cerebrovasculature in Alzheimer's disease (AD), causes a dramatic immune response that prevents plaque formation and clears accumulated Aβ in transgenic mice. In a clinical trial of Aβ immunization, some patients developed meningoencephalitis and hemorrhages. Neuropathological investigations of patients who died after the trial showed clearance of amyloid pathology, but also a powerful immune response involving activated T cells probably underlying the negative effects of the immunization.

**Results:**

To define the impact of T cells on this inflammatory response we used passive immunization and adoptive transfer to separate the effect of IgG and T cell mediated effects on microhemorrhage in APPPS1 transgenic mice. Neither anti Aβ IgG nor adoptively transferred T cells, alone, led to increased cerebrovascular damage. However, the combination of adoptively transferred T cells and passive immunization led to massive cerebrovascular bleeding that ranged from multiple microhemorrhages in the parenchyma to large hematomas.

**Conclusions:**

Our results indicate that vaccination can lead to Aβ and T cell induced cerebral micro-hemorrhages and acute hematomas, which are greatly exacerbated by T cell mediated activity.

## Background

Immunotherapeutic approaches to treat neurodegenerative diseases have gained prominence in the last few years, particularly in the field of Alzheimer's disease (AD) research. AD and mouse models of amyloid pathology both have an innate immune response to amyloid-β deposition, which is probably beneficial [1]. The devastating mental effects of Alzheimer's disease are caused by pathological changes in the brain including the accumulation of amyloid-β peptide (Aβ) either in the parenchyma in the form of senile plaques or in the cerebrovasculature (CAA-cerebral amyloid angiopathy). Mice that have been genetically altered so that they have quantities of Aβ in their brains have plaque pathology similar to that seen in Alzheimer's patients [2-4]. Studies of immunizing these APP transgenic mice with the Aβ peptide led to the exciting discovery that the immune system can prevent plaque formation and even reverse some of the damage by producing antibodies to Aβ [5]. Subsequent experiments confirmed the neuropathological benefits of immunization and showed that it also benefits the behavioral symptoms associated with amyloid deposition [6-10]. Furthermore the clearance of plaques can lead to normalization of the neuritic processes and dystrophies surrounding plaques, suggesting an astonishing degree of plasticity in the adult brain, and potential for recovery [11-13].

Based on this evidence in APP transgenic mice that immunization can reduce Alzheimer's pathology, a clinical trial was run in which patients with Alzheimer's disease were immunized against Aβ. A Phase I study in AD patients demonstrated good safety and tolerability of multiple injections of aggregated Aβ42 (AN 1792) [14]. Clinical evaluation showed slower rates of decline of mental function in some of the treated patients [15], but other analyses showed that the clinical effect was at most minimal [14,16]. A postmortem examination of this Phase I immunization trial showed evidence of a significant reduction in brain amyloid plaques, together with increased blood anti-Aβ antibody levels [17]. However, the Phase II trial was discontinued after 6% of the participants developed meningoencephalitis, a life-threatening inflammatory reaction [18]. Two patients died and examination of their brains showed decreased amounts of plaques when compared to nonimmunized patients, but there was also evidence of inflammatory infiltrates that involved activated T cells [16,19].

To circumvent these adverse effects, another Phase 2 trial of passive immunization with the N-terminal monoclonal antibody Bapineuzumab was carried out. Although Bapineuzumab did not offer a clinical benefit, a subgroup of ApoE4 non-carriers performed better on cognitive measures and experienced fewer adverse effects. The most concerning adverse effect of this study was the development of vasogenic oedema, which occurred in 10% of the patients preferentially ApoE4 carriers treated with higher doses of ≥ 1 mg/kg Bapineuzumab [20,21]. In a smaller Phase 2 trial, PET imaging with the amyloid tracer Pittsburgh Compound B showed a reduction in brain amyloid load in AD patients treated with Bapineuzumab. In agreement with the above mentioned study, 2 patients receiving the higher dose of 2 mg/kg developed cerebral vasogenic oedema [22].

Understanding the factors that are required to induce Aβ encephalitis and microhemorrhages is crucial for the development of Aβ vaccination approaches. In order to address the important clinical question of why immunization against Aβ causes a harmful inflammatory reaction in the brain we monitored the T cell response to Aβ immunization in APPPS1 transgenic mice. Transgenic mice expressing green fluorescent protein (GFP) in specific subpopulations of T cells [23] were immunized with Aβ42 followed by adoptive transfer of these fluorescently labeled T cells into APPPS1 transgenic mice. With our experimental setting we observed microhemorrhages in the host mice after application of anti Aβ antibodies, similar to those observed in human patients; in at least one patient, frank hemorrhage was also reported [19], similar to the results in this animal model. These results suggest that Aβ activated T cells would be a possible marker to screen for in AD patients before they undergo passive immunization.

## Results

### Generation of Aβ specific T cells ex vivo

**Figure 1:**
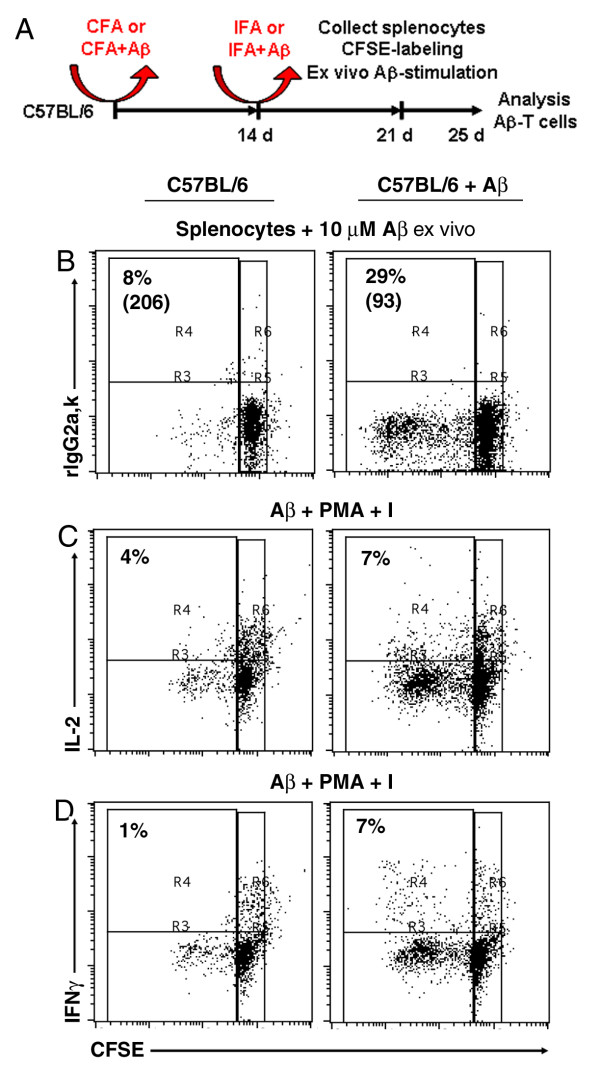
**Generation of Aβ-specific effector T cells**. (**A**) Thy1.1+ C57Bl/6 mice were immunized subcutaneously with Aβ peptide plus CFA or with CFA alone (control mice). 14 days later, the mice were boosted with either Aβ peptide plus IFA or IFA alone. 7 days later the mice were euthanized and splenocytes were isolated from either Aβ-immunized or control mice. (**B-D**) In order to assess Aβ-specific T cell responses, CFSE-labeled splenocytes were incubated ex vivo with Aβ peptide and 4 days later were analyzed for their CFSE dilution (as a readout of T- cell proliferation) (**B**) and production of effector cytokines by intracellular staining (**C and D**). Flow cytometry analyses were restricted to viable cells and gated on T cells. T cells from mice immunized with Aβ peptide exhibited a higher degree of proliferation (CFSE dilution) and produced higher levels of effector cytokines when restimulated *ex vivo *with Aβ peptide as compared to T cells from control mice.

Thy1.1+ congenic mice were immunized subcutaneously with either Aβ peptide plus complete Freund`s adjuvant (CFA) or with CFA alone and boosted after 14 days. At day 21 total splenocytes were re-stimulated *ex vivo *with Aβ peptide and the T cells were analyzed for their degree of proliferation with the fluorescent dye 5,6-carboxylfluorescein diacetate succinimidyl ester (CFSE dilution) and production of effector cytokines (Figure 1). As expected, T cells from mice previously immunized with Aβ peptide exhibited a higher proliferative capacity (Figure 1B) and produced higher levels of effector cytokines such as IL-2 and IFNγ as compared to T cells from control mice (Figure 1C,D). These results indicate that Aβ immunization was effective for priming and expanding Aβ-specific effector T cells.

### Development of acute hematomas

**Figure 2:**
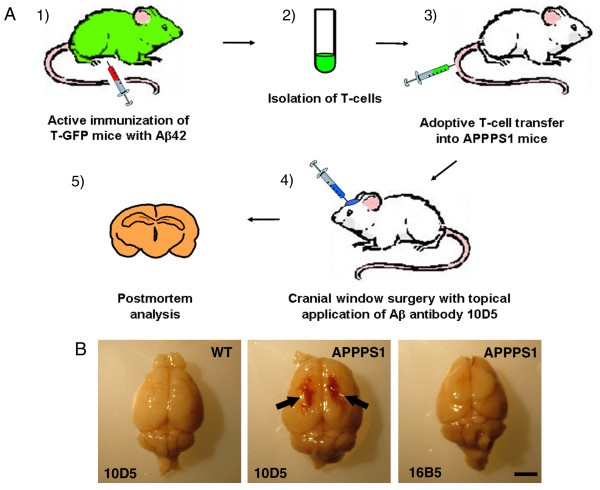
**Schematic illustration of the experimental in vivo approach and the incidence of bleedings in APPPS1 transgenic mice**. (**A**) We immunized T-GFP mice with Aβ42 and adoptively transferred these isolated T cells via the tail vein into transgenic or control mice. After a recovery period of one week, a cranial window surgery with dura removal and antibody application of either antibody 10D5 against Aβ or control antibody 16D5 against human tau was performed. Brains were analyzed postmortem 3 to 4 days after passive immunization. (**B**) In contrast to wildtype mice passively immunized (left image) or APPPS1 transgenic mice immunized with a control antibody against human tau (right image) transgenic mice immunized with anti Aβ antibody developed acute hematomas on both hemispheres as indicated by black arrows (image in the middle).

With age, APPPS1 transgenic mice develop dense core Aβ deposits in the parenchyma in forms of plaques and, later and in small amounts, around blood vessels (CAA). In order to investigate the T cell inflammatory response, developed as a consequence of active immunization in human Alzheimer's patients, we immunized T-GFP mice with Aβ42 followed by adoptive transfer of these fluorescently labeled T cells into mice that are transgenic for human APP. One week after the adoptive transfer, we performed a cranial window surgery with dura removal and antibody application of either antibody 10D5 against Aβ or control antibody 16D5 against human tau (Figure 2A). In vivo multiphoton microscopy for visualization and tracking of T cells in the brain of APPPS1 transgenic mice did not detect any transferred T cells during 1 hour in vivo imaging sessions (Additional File 1, Figure S1 A,B) although green T cells were readily found at post mortem examination, most prominently in the spleens of the mice (Additional File 1, Figure S1 D).

Instead, after adoptive transfer of Aβ activated T cells we observed bleeding and hemorrhage in APPPS1 transgenic mice after T cell transfer and immunization procedure (Figure 2B) similar in many respects to those reported in human patients undergoing active immunization. All APPPS1 transgenic animals that received both T cell transfer and Aβ antibody treatment developed massive bleeding 3-4 days after the surgical procedure and passive immunization in contrast to the wildtype animals. In control experiments where either the control antibody 16B5 or no antibody was administered or where only the antibody 10D5 was applied but no GFP positive T cells transferred, no sign of bleeding was evident in the mice, suggesting that the bleeding was probably due to a combination of both Aβ-specific antibody application and adoptive T cell transfer.

### Adoptive transfer of primed T cells exacerbates microhemorrhages after passive immunization in APPPS1 transgenic

**Figure 3:**
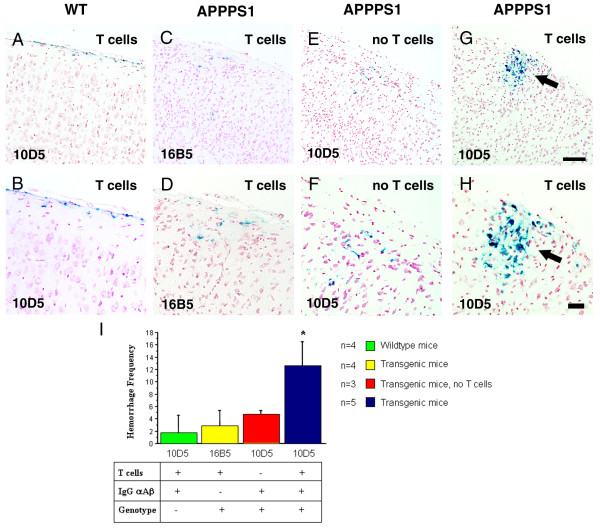
**Increase in number of old microhemorrhages following passive immunization**. (**A-H**) Sections are stained for hemosiderin (extravenous iron in blue) with Prussian Blue and cells counterstained with nuclear fast red (**A-G**) Panels show the cortex of either a wildtype control mouse **(A)**, APPPS1 transgenic mouse treated with control antibody 16B5 **(C) **or 10D5 treated APPPS1 mouse lacking the transferred T cells **(E)**. The arrow in **(G) **denotes a massive clustering of hemosiderin positive microglia in T cell transferred mice following IgG αAβ treatment. **(B-H) **Panels are a higher magnification of the cortical area shown in **(A-G)**. (**I**) Quantification of Prussian Blue staining illustrated as the number of positive profiles per brain. Note the fourfold increase in hemorrhage frequency in APPPS1 transgenic mice which received T cells and were passively immunized. Scale bars, 100 μm (A,C,E,G), 50 μm (B,D,F,H).

**Figure 4:**
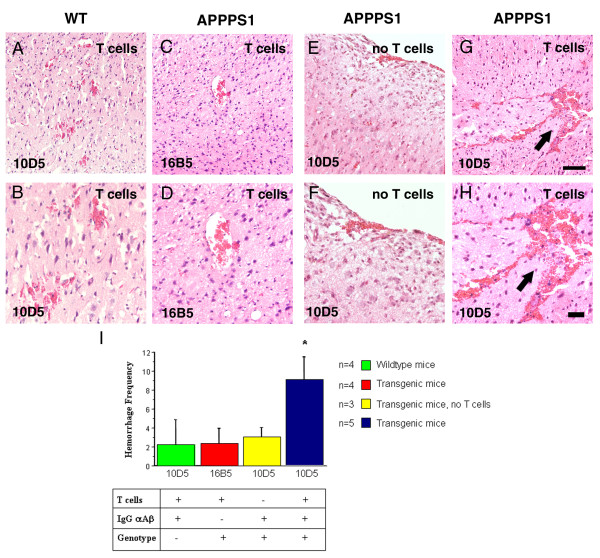
**Increase in number of acute hematomas following passive immunization**. (**A**-**H**) Evidence of acute hematomas was assessed in H&E-stained sections confirming the occurrence of acute bleedings in all animals used in this study. **(G) **The black arrow indicates a significant hemorrhage in T cell transferred mice following IgG αAβ treatment. **(B-H) **Panels are a higher magnification of the cortical area shown in **(A-G)**. **(I) **Quantification of acute hemorrhages in the different groups revealed a threefold increase in APPPS1 transgenic mice that have received Aβ primed T cells and were passively immunized. Scale bars, 100 μm (A,C,E,G), 50 μm (B,D,F,H).

Due to the massive bleeding, it was not possible to reimage these animals and therefore the brains were analyzed postmortem. It has been previously reported that APP transgenic mice that received passive anti-Aβ immunotherapy develop CAA-associated microhemorrhages [24-26]. To determine whether transferred T cells induced additional microhemorrhages after passive immunization we carried out microhemorrhage analysis. Old hemorrhages were studied using Prussian Blue stain, which identifies residual hemosiderin. Acute bleeding was assessed in H&E-stained sections. We detected hemosiderin deposits in the cortex that were localized in the cytoplasma of microglial cells in all treated mice (Figure 3A-H). Strikingly, we found a dramatic fourfold increase in the number of microhemorrhages specifically in the cortex of APPPS1 transgenic mice that received T cells and were passively immunized. In addition, it is important to note that these old microhemorrhages appeared to be also larger in size than the rare microhemorrhages seen in animals that did not receive T cells (Figure 3G,H,I). Similar results were obtained when acute hemorrhages were analyzed with an approximately threefold increase in microhemorrhage frequency as well as increase in hemorrhage size (Figure 4).

### T cell related microhemorrhages segregated from CAA

The distribution of the microhemorrhages in the cortex of 10D5-treated mice was consistent with the distribution of CAA reported in this APPPS1 transgenic mouse model [27]. To determine if microhemorrhages were directly associated with CAA, we additionally stained the sections for deposited amyloid by Thioflavine-S. In agreement with an earlier study these APPPS1 transgenic mice developed only very limited amount of CAA [27]. We did not find CAA-associated microhemorrhages but instead we observed microhemorrhages that were not closely related to CAA (Additional File 1, Figure S2 A-H).

**Figure 5:**
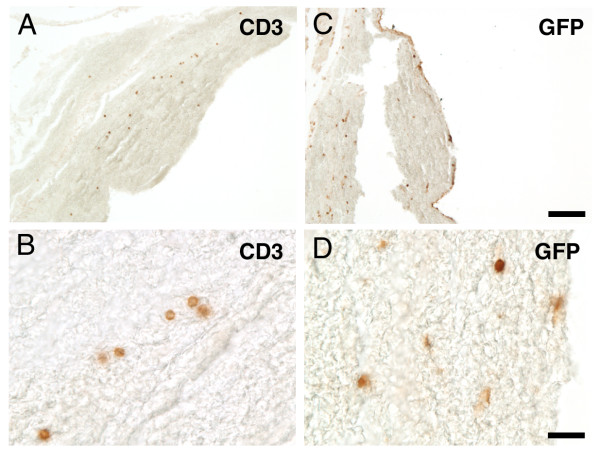
**GFP and CD3 positive T cells infiltrate the CNS and are mainly located within the hemorrhages**. **(A,B) **CD3 immunostaining of the cortex revealed a cluster of sporadic T cells throughout a significant hemorrhage. **(C,D) **GFP immunostaining of an adjacent section of the same area confirmed the occurrence of Aβ primed adoptively transferred T cells restricted to this hematoma. Scale bars, 100 μm (A,B), 20 μm (C,D).

To investigate whether these induced microhemorrhages may have been caused by the infiltration of Aβ-specific T cells into the CNS, we immunostained brain sections with anti-GFP and anti-CD3 antibodies to identify all transferred T cells in the parenchyma. We observed a few clusters of T cells positive for CD3 (Figure 5A,B) as well as for GFP (Figure 5C,D) primarily located within the acute hemorrhages, indicating that the adoptively transferred T cells indeed entered the CNS and secondly caused massive bleeding. Notably, these few small groups of CD3 positive cells were not associated with plaques. Likewise, we did not observe any CD3 positive cell clustering around the vasculature, which would have been expected to occur with an encephalitogenic response.

## Discussion

To continue developing clinical AD therapies based on Aβ immunization, such strategies will clearly need to avoid meningoencephalitic reactions to Aβ immunizations previously observed in humans [1,18]. Postmortem analysis suggested that inflammatory infiltrates found in the AD brain involved activated T cells [16,19]. Here we investigated in an animal model the impact of T cells (previously exposed to the antigen) on the inflammatory events that occur during Aβ immunization by transferring activated T cells of TGFP mice into APPPS1 transgenic mice following anti Aβ antibody application in order to separate the effect of IgG and T cell mediated effects. The infusion of Aβ specific T cells (using a protocol enriching for Th2 T cells, in comparison to our protocol) into APPPS1 transgenic mice has previously been shown to have beneficial effects such as reversing cognitive impairment and synaptic loss without infiltrating the CNS [28,29]. Extending these experimental procedures we performed passive immunization of the recipient APPPS1 mice and found massive bleeding and microhemorrhages, delayed several days after the infusion of activated T cells, indicating that the combined effect of activated T cells and passive immunization procedures can be devastating. Whether transferred T cells crossed the blood brain barrier and were attracted by senile plaques or CAA could not be inferred from these experiments, because massive bleeding started 3 days after surgery and made live imaging with 2-Photon microscopy impossible. We therefore were restricted methodologically to postmortem brain analysis in addressing T cell-mediated inflammatory events that occur during amyloid-β (Aβ) immunization. In this study, we did not observe the previously reported correlation between microhemorrhages and CAA in APP transgenic mice [24-26,30,31]. This might be due to the fact that the mouse model we used only develops a limited amount of CAA in general [27].

T cell trafficking into the CNS is thought to occur when activated T cells cross the blood brain barrier. One can think of 2 possible scenarios: T cell mediated neuropathological events may occur indirectly as a result of bystander damage by co-infiltrating T cells with irrelevant antigen specificities or may be mediated directly by antigen specific T cells. Our data speak for the latter insofar as only transferred T cells previously stimulated by Aβ42 (in combination with antibody treatment of the recipient mouse) induced frank bleeding or microhemorrhages. We indeed observed cells positive for GFP and CD3 located within the bleeding, showing that these antigen specific T cells crossed the blood brain barrier and likely participate to the undesirable side effect of Aβ immunization. This result is in accord with a study demonstrating that bystander T cells can only traffic to the CNS with very limited efficiency and have only small contribution to neuropathological changes [32].

The fact that only the combination of both T cells and Aβ antibodies induced microhemorrhage indicates that reactive T cells could be a potential biomarker to screen for higher risk AD patients considering passive immunization against Aβ. The goal of safer immunotherapy approaches is already under development, although vasogenic edema has been observed even with only passive immunization, and current active anti-Aβ vaccine designs do not include a self T cell epitope.

## Conclusions

In sum, our results provide two key implications: first, in this model it appears that T cells participate in the pathophysiological process associated with anti-Aβ immune mediated microhemorrhage. Second, although immunization-associated microhemorrhage has been reported in a large number of mouse models [24-26,30,33], the microhemorrhages are frequently inconsistent and scattered; because the current data suggest uniform readily detected microhemorrhage within a small time window of antibody therapy, the current protocol may provide a more systematic method to test various anti-Aβ therapeutics for hemorrhagic effects of immunotherapy.

## Methods

### Animals

APPswe/PS1d9 transgenic mice aged 7-11 months and control littermates the same age were used in this study (obtained from Jackson lab, Bar Harbor, Maine) [4]. APPswe/PS1d9 transgenic mice start to develop plaques and CAA in the neocortex between 5 and 7 months of age. TGFP transgenic mice that express green fluorescent protein (GFP) under the control of the murine CD4 promoter in CD4^+ ^and CD8^+ ^have been used as donor for the adoptive transfer experiments [34] (obtained from Uli von Andrian). All animal work was conducted with approved protocol from the Massachusetts General Hospital Animal Care and Use Committee and in compliance with NIH guidelines for the use of experimental animals.

#### T cell isolation and adoptive T cell transfer

Thy1.1+ C57BL/6 mice or DPE-T-GFP mice (in which all T cells express GFP) [34] were immunized subcutaneously with Aβ42 peptide (2 mg/mouse) in complete Freund's adjuvant (CFA, Sigma-Aldrich, Saint Louis, MO). The concentration of mycobacteria in the CFA formulation was 0,1%. 14 days later, the mice were boosted with 2 mg Aβ peptide in incomplete Freund's adjuvant (IFA). Control mice were immunized and boosted only with CFA and IFA, respectively. 1 week later (day 21) the mice were euthanized and the splenocytes were labeled with 5 μM carboxyfluorescein succinimidyl ester (CFSE) and cultured *ex vivo *with or without 10 μM Aβ peptide. T cells were analyzed by flow cytometry at day 4 for their CFSE dilution (T cell proliferation) and cytokine production (IL-2 and IFNγ) by intracellular staining. In order to detect intracellular cytokines, T cells were additionally re-stimulated for 4 hours with PMA plus ionomycin in the presence of brefeldin-A, as described [35]. T cells of immunized DPE-T-GFP mice (140 × 10^7 ^cells in 0.3 ml total volume) were adoptively transferred via the tail vein into recipient APPPS1 transgenic or non transgenic control mice.

#### Surgical procedure

One week after the adoptive transfer of T cells, mice received an intraperitoneal injection of methoxy-O4 (5 mg/kg) a fluorescent compound that crosses the blood-brain barrier and binds to amyloid plaques and CAA [36]. The following day a cranial window surgery was done under avertin anesthesia (1.3% 2,2,2-tribromethanol, 0.8%tert-pentylalcohol; 250 mg/kg) by installing a glass window 6 mm in diameter on top of the brain [36-38]. The dura was carefully retracted to the midline with fine forceps and a single antibody application of 20 μl of 1 mg/ml of either an antibody against the N-terminal of Aβ (10D5, IgG1) or a control antibody directed to an unrelated epitope (anti-human tau 16B5) was then applied to the surface of the brain before attachment the glass coverslip with dental cement. Texas Red dextran (70,000 Daltons molecular weight, 12.5 mg/ml in sterile phosphate-buffered saline, Molecular probes, Eugene, OR) was injected into the lateral tail vein to provide an angiogram.

#### Imaging methods

Mice were imaged immediately after surgery while still under anesthesia, and then allowed to recover. *In vivo *imaging of amyloid pathology and blood vessels in the living mouse brain was performed on a Bio-Rad 1024ES multiphoton microscope (Bio-Rad Laboratories, Hercules, CA), mounted on an Olympus BX50WI upright microscope (Olympus Optical, Tokyo, Japan). A wax ring was placed around the coverslip of the cortical window and filled with distilled water to create a well for an Olympus 20x dipping objective (numerical aperture 0.95). A mode-locked Ti:Saphire laser (MaiTai, Spectra-Physics, Mountain View, CA) generated two-photon fluorescence with 800 nm excitation, and detectors containing three photomultiplier tubes (Hamamatsu Photonics, Bridgewater, NJ) collected emitted light in the range of 380-480, 500-540, and 560-650 nm. During each imaging session, images at low resolution (615 μm × 615 μm × 5 μm sections; 10-20 sections per stack) were captured to provide an overview of the area, followed by images with high resolution (approximately 150 μm × 150 μm × 1 μm sections; 15-50 sections per stack) to zoom in on a specific area. At the end of each imaging sessions, mice were allowed to recover on a heating pad and placed singly in their home cage.

#### Postmortem analysis

3 to 4 days after 10D5 antibody application for passive immunization, the mice developed hematomas and therefore had to be sacrificed immediately. Mice were euthanized by CO_2 _inhalation and the brains and spleens were immersion-fixed for 2 d in 4% paraformaldehyde, and the brains then embedded in paraffin. Coronal serial sections of 8 μm thickness were cut with a microtome throughout the brain. Hematoxylin and eosin (H&E) and Thioflavine-S staining were done according to standard protocols [39] and Prussian Blue staining was performed as previously described [39,40]. The Prussian Blue method was used to visualize old hemorrhages by microglia that have been engulfed ferric iron-containing hemosiderin from red blood cells. To test if microhemorrhages were associated with CAA, some sections were costained with Thioflavine-S. Prussian Blue stain was imaged first because of extremely rapid dissolution of the blue precipitate after UV excitation required for Thioflavine-S imaging. Immunohistochemistry on paraffin sections was performed by using the avidin-biotin-peroxidase complex method (Vector Laboratories, Burlingame, CA) with diaminobenzidine as chromogen. The following primary antibodies were used: polyclonal antibody against GFP (Abcam, Cambridge, MA) and the rat monoclonal antibody against CD3 (AbD Serotec, Raleigh, NC). Both stainings were done with heat-induced antigen retrieval as recommended by the antibody providers (citrate buffer, pH 6.0 at 95°C for 20 min). Micrographs of immunostaining were obtained on an upright Olympus Optical BX51 fluorescence microscope with an Olympus Optical DP70 camera, and images were arranged in Adobe Photoshop (Adobe Systems, San Jose, CA).

#### Quantification

Cerebral hemorrhage is accompanied by a delayed appearance of hemosiderin-positive microglia [41]. Prussian blue stained clusters of hemosiderin staining was quantified on sets of systematically sampled sections (every 10^th ^section throughout the cortex). An additional set of every 10^th ^section was stained for H&E and screened for acute intraparenchymal bleedings visible as large accumulation of erythrocytes in the parenchyma.

#### Statistical analysis

All quantitative analysis was performed without knowledge of the treatment group. Two independent experimenters counted the microhemorrhages, and an average score was used. All statistical analyses were done with the StatView program (SAS Institute, Inc, Cary, NC). Data were expressed as mean ± standard deviation from the mean. Significance levels were set at p < 0.05.

## Competing interests

The authors declare that they have no competing interests.

## Authors' contributions

MML contributed to the general design of the experiments, performed and supervised the experiments and helped to draft the manuscript. JRM isolated, prepared and characterized the T cells for adoptive transfer. MM performed immunostaining experiments and quantified microhemorrhages. TLSJ and ADC helped and performed in vivo experiments of adoptive T cell transfer. UVA contributed to the design and interpretation of the study and review of the manuscript. BTH designed and supervised all experiments, contributed to data interpretation and manuscript preparation. All authors have read and approved the manuscript.

## Supplementary Material

Additional file 1 **Figure S1: GFP positive T cells could not be detected in the brain via multiphoton imaging**. **(A,B) **Three-colour in vivo multiphoton images showing senile plaques in blue along with blood vessels in red from the living brain of an APPPS1 transgenic mouse that previously received Aβ specific GFP positive T cells. No evidence of GFP positive T cells was observed. **(C) **In contrast, many green T cells could be detected in the spleen but were missing in the spleen of a control mouse which did not receive GFP positive T cells **(D)**. Scale bars, 100 μm (A,B), 100 μm (C,D). **Figure S2: Microhemorrhages are not related to CAA (A,B) **Prussian Blue stained sections show clustering of hemosiderin positive microglia in the cortex (black arrow). **(C,D) **The white arrowhead points towards CAA located away from the bleeding in Thioflavine-S stained consecutive sections. **(E,F) **Another example of a Prussian Blue positive microhemorrhage without any Thioflavine-S positive CAA in its vicinity **(G,H)**. Scale bars, 400 μm (A,B), 100 μm (C,D). Click here for file
