# Supplementary figures and images for: T cell mediated cerebral hemorrhages and microhemorrhages during passive Aβ immunization in APPPS1 transgenic mice
Source: Mol Neurodegener. 2011 Mar 9;6:22. doi: 10.1186/1750-1326-6-22 (PMC3068114; doi:10.1186/1750-1326-6-22)

**
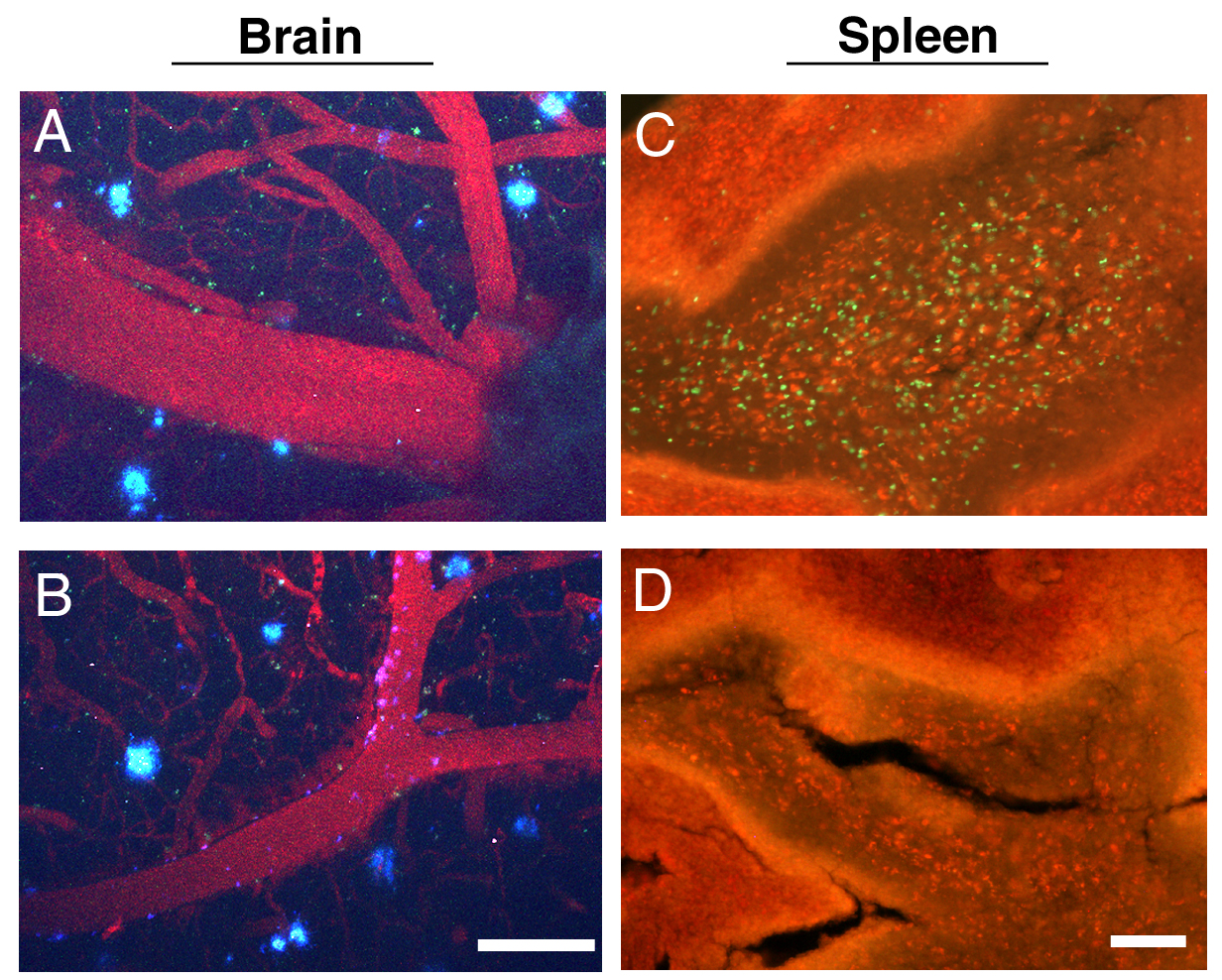
**

**Additional File 1, Figure S1**

**
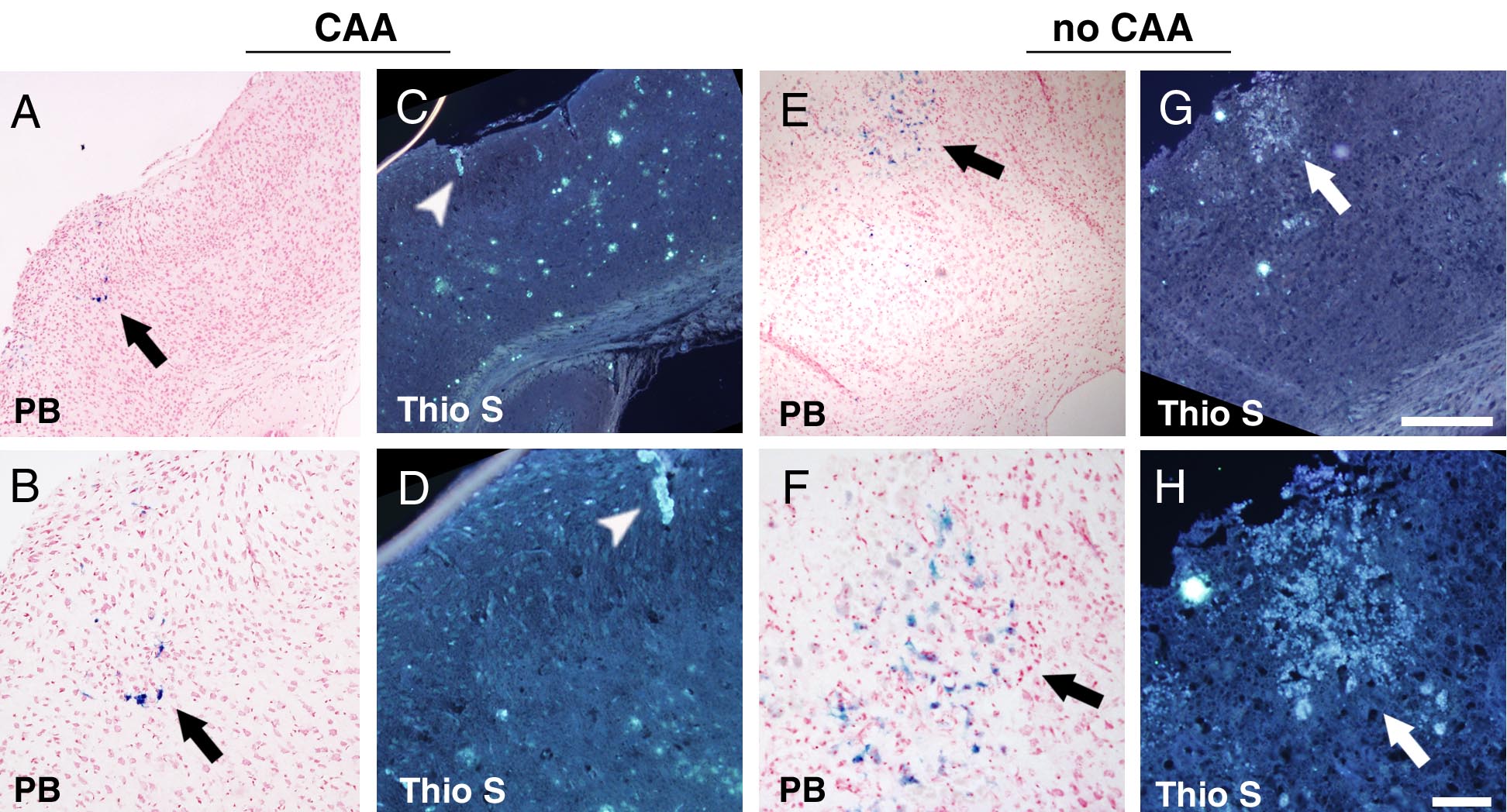
**

**Additional File 1, Figure S2**

Supplement: Additional file 1 — Figure S1: GFP positive T cells could not be detected in the brain via multiphoton imaging. (A,B) Three-colour in vivo multiphoton images showing senile plaques in blue along with blood vessels in red from the living brain of an APPPS1 transgenic mouse that previously received Aβ specific GFP positive T cells. No evidence of GFP positive T cells was observed. (C) In contrast, many green T cells could be detected in the spleen but were missing in the spleen of a control mouse which did not receive GFP positive T cells (D). Scale bars, 100 μm (A,B), 100 μm (C,D). Figure S2: Microhemorrhages are not related to CAA (A,B) Prussian Blue stained sections show clustering of hemosiderin positive microglia in the cortex (black arrow). (C,D) The white arrowhead points towards CAA located away from the bleeding in Thioflavine-S stained consecutive sections. (E,F) Another example of a Prussian Blue positive microhemorrhage without any Thioflavine-S positive CAA in its vicinity (G,H). Scale bars, 400 μm (A,B), 100 μm (C,D). [file 1750-1326-6-22-S1.DOC]
